# Transcriptome of macrolide-resistant *emm92* invasive group A *Streptococcus* in response to erythromycin exposure

**DOI:** 10.1128/mra.00840-25

**Published:** 2025-11-06

**Authors:** Lillie M. Powell, Soo Jeon Choi, Lei Wang, Gangqing Hu, F. Heath Damron, Slawomir Lukomski

**Affiliations:** 1 Department of Microbiology, Immunology, and Cell Biology, West Virginia University School of Medicine 12355, Morgantown, West Virginia, USA; 2 Vaccine Development Center at West Virginia University Health Sciences Center 53422 , Morgantown, West Virginia, USA; 3 West Virginia Clinical and Translational Institute, West Virginia University School of Medicine 12355, Morgantown, West Virginia, USA; University of Maryland School of Medicine, Baltimore, Maryland, USA

**Keywords:** group A *Streptococcus*, *emm92*, transcriptome, erythromycin

## Abstract

Macrolide-resistant *emm92*-type invasive *Streptococcus pyogenes* has emerged in the United States since 2010, posing the question of whether acquired *erm*(T)-encoded resistance may have contributed to the rise in infections. Here, we present the transcriptomes for two *Streptococcus pyogenes* isolates of *emm*-type 92 in response to erythromycin exposure.

## ANNOUNCEMENT

Circa 2010, an MLS_B_ (macrolide, lincosamide, streptogramin B) resistant *emm92* strain of *Streptococcus pyogenes* (e.g., group A *Streptococcus* or GAS), harboring the *erm*(T) gene, emerged in the U.S. as a major cause of invasive infection (1–6). We previously reported differences in *erm*(T)-gene transcription between *emm92* isolates displaying an inducible (iMLS_B_) or constitutive (cMLS_B_) resistance phenotype. When isolates were exposed to erythromycin, we observed delayed *in vitro* growth of iMLS_B_ isolates and a higher clindamycin MIC in cMLS_B_ isolates (7). Here, we investigated how antibiotic exposure impacts the global *emm92* transcriptome.

**Fig 1:**
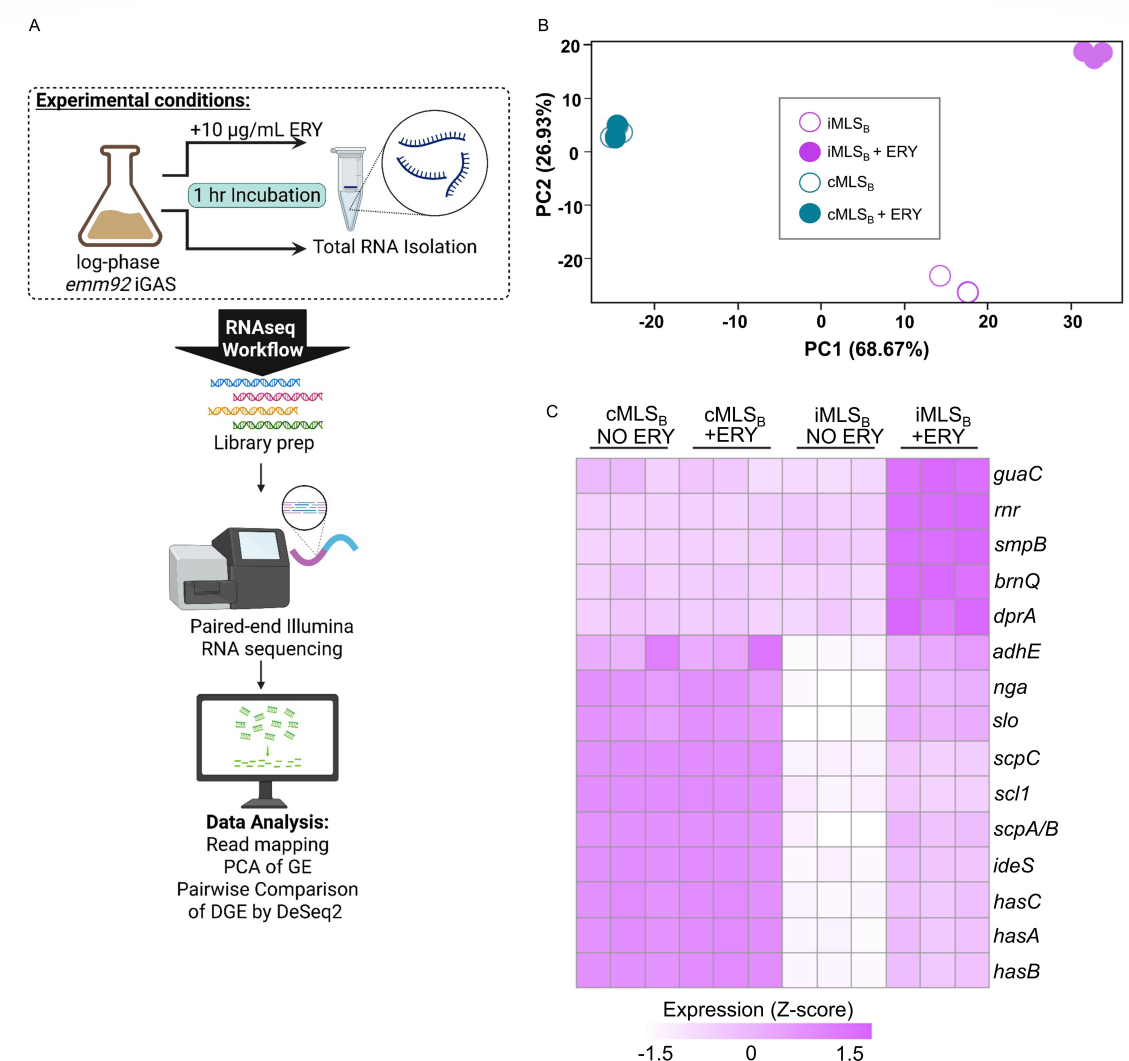
(**A**) Schematic of experimental conditions for iGAS culture, subsequent RNA sequencing, and analysis; panel generated with BioRender.com. (**B**) Principal component analysis based on whole genome gene expression (measured as RPKM) for samples corresponding to an *emm92* iMLS_B_ isolate WVGAS15 (pink) or cMLS_B_ isolate WVGAS10 (teal) in the absence or presence of 10 µg/mL erythromycin (ERY). Three biological replicates are shown for each isolate and condition. The percentage of variance explained by the first two principal components was calculated using MeV (13). (**C**) Heatmap visualization of gene expression values for 15 DE genes (rows), annotated with known functions, was identified by analysis of isolates in media without (no ERY) or with (+ERY) erythromycin according to MLS_B_ sub-phenotype. Each column represents a sample from independent experiments. The output of the DESeq2 analysis is available under GEO GSE303398. The expression values for each gene were z-transformed across samples (rows).

Isolates from the state of West Virginia (i) WVGAS10 (cMLS_B_ phenotype) from a mediastinal abscess and (ii) WVGAS15 (iMLS_B_ phenotype) from an antecubital fossa abscess (4) were arbitrarily selected for this study. Isolates were cultured in Todd Hewitt Yeast media at a starting OD_600nm_ of 0.05 and incubated at 37°C with 5% CO_2_ (Fig. 1A). At log phase (OD_600nm_ 0.5), 10 µg/mL of erythromycin was added to half of each culture and incubated for 1 hour. Total RNA was isolated from three independent experiments using the rBAC Mini Total RNA kit (IBI Scientific, Dubuque, IA) per manufacturer’s instructions for Gram-positive bacteria. DNase I digestion was performed with a TURBO DNA-free kit from Invitrogen (Thermo Fisher Scientific, Waltham, MA). Sample quality was assessed via Agilent tape station by Admera Health (Plainfield, NJ) (8). Illumina kits were used for cDNA library generation (QIAseq FastSelect rRNA 5S/16S/23S [Bacteria] kit and NEB Ultra II Directional RNA Library Prep kit). RNA sequencing was performed by Admera Health on the Illumina NovaSeq X platform with paired-end sequencing (2 × 150 base pairs) (Table 1), at a depth of 60 million reads per sample (New England Biolabs, Ipswich, MA). Data were analyzed using the QIAGEN CLC Genomics Workbench version 23 RNA-seq analysis pipeline with default settings (QIAGEN, Aarhus, Denmark) (9). Reads were trimmed and mapped to the MGAS2221 genome (CP043530.1). Differentially expressed (DE) gene analysis, performed with DESeq2 (10–12), for erythromycin-treated versus untreated conditions, was independently conducted for each MLS_B_ sub-phenotype, resulting in two pairwise comparisons (Table 1). Statistical significance of DE genes was determined according to an adjusted false discovery rate *P* value of <0.05 and a log_2_ fold-change >1.5. The Z-transformation was applied when visualizing gene expression values across samples for DE genes. Default parameters were used for all software except where otherwise noted (Table 1).

**TABLE 1:** Summary of RNA sequencing sample characteristics^*a*^^,*b*,*c*^

Sample	*emm92*-type strains of group A *Streptococcus*	MLS_B_ phenotype	Treatment conditions	Replicate	GEO accession no.	SRA accession no.
1	WVGAS10	cMLS_B_	No erythromycin/1 h	1	GSM9125281	SRX30658464
2	WVGAS10	cMLS_B_	No erythromycin/1 h	2	GSM9125282	SRX30658465
3	WVGAS10	cMLS_B_	No erythromycin/1 h	3	GSM9125283	SRX30658468
4	WVGAS10	cMLS_B_	10 µg/mL erythromycin/1 h	1	GSM9125284	SRX30658469
5	WVGAS10	cMLS_B_	10 µg/mL erythromycin/1 h	2	GSM9125285	SRX30658470
6	WVGAS10	cMLS_B_	10 µg/mL erythromycin/1 h	3	GSM9125286	SRX30658471
7	WVGAS15	iMLS_B_	No erythromycin/1 h	1	GSM9125287	SRX30658472
8	WVGAS15	iMLS_B_	No erythromycin/1 h	2	GSM9125288	SRX30658473
9	WVGAS15	iMLS_B_	No erythromycin/1 h	3	GSM9125289	SRX30658474
10	WVGAS15	iMLS_B_	10 µg/mL erythromycin/1 h	1	GSM9125290	SRX30658475
11	WVGAS15	iMLS_B_	10 µg/mL erythromycin/1 h	2	GSM9125291	SRX30658466
12	WVGAS15	iMLS_B_	10 µg/mL erythromycin/1 h	3	GSM9125292	SRX30658467

^*a*^ Default settings in the CLC genomics workbench version 23 RNA-seq analysis pipeline were used to analyze transcriptomic data unless otherwise noted. Mapping to the MGAS221 genome (CP043530.1) was performed with the CLC Mapper tool (parameters were as follows: mismatch cost: 2, insertion cost: 3, deletion cost: 3, length fraction: 0.8, similarity fraction: 0.8).

^*b*^ To assess differential gene expression by DESeq2, the CLC Genomics Workbench version 23 was used. This proprietary software employs a generalized linear model with a negative binomial distribution similar to as described previously 10–12.

^*c*^ The design matrix used to determine differential expression was two independent pairwise comparisons of the following samples: [comparison 1] WVGAS10_NO_ERY (samples 1–3) versus WVGAS10_ERY (samples 4–6), [comparison 2] WVGAS15_NO_ERY (samples 7–9) versus WVGAS15_ERY (samples 10–12). For comparison of expression values across all samples, gene expression values for DE genes were visualized by z-transformation.

Principal component analysis with MeV (13) identified transcriptomic differences in total gene reads following exposure to erythromycin (ERY) between the iMLS_B_, but not the cMLS_B_ samples (Fig. 1B). For DE analysis, ERY exposure did not change expression at the individual gene level for the cMLS_B_ samples. In contrast, we identified 48 upregulated genes for the iMLS_B_ samples. We visualized the expression of 15 such genes with function annotation across all samples (Fig. 1C), which included those encoding key GAS pathogenicity factors, such as *hasA-C*, *ideS*, *scpA*, *scl1*, *scpC*, and *slo* (14–20). The *emm92* transcriptome data set provides a resource to elucidate how erythromycin/macrolide treatment may have supported the emergence and pathogenesis of the *erm*(T)-harboring *emm92* strain.

## Data Availability

The data sets for raw sequencing data, gene counts, and DESeq2 results are available on the GEO database under accession GSE303398 and on the SRA database under accession PRJNA1182229. Isolates used in this study can be made available for research upon request via an MTA agreement.
